# An Optimized SVR Algorithm for Pulse Pile-Up Correction in Pulse Shape Discrimination

**DOI:** 10.3390/s24237545

**Published:** 2024-11-26

**Authors:** Xianghe Liu, Bingqi Liu, Mingzhe Liu, Yufeng Tang, Haonan Li, Yao Huang

**Affiliations:** 1 The College Nuclear Technology and Automation Engineering, Chengdu University of Technology, Chengdu 610059, China; liuxianghe1@cdut.edu.cn; 2 School of Data Science and Artificial Intelligence, Wenzhou University of Technology, Wenzhou 325000, China; liubingqi@cdu.edu.cn; 3 School of Mechanical Engineering, Chengdu University, Chengdu 610106, China; tangyufeng@stu.cdu.edu.cn (Y.T.); lihaonan@stu.cdu.edu.cn (H.L.); 4 School of Medicine, Chongqing University, Chongqing 400044, China; huangyao@stu.cqu.edu.cn

**Keywords:** pile-up pulse, support vector regression, pulse shape discrimination

## Abstract

Pulse pile-up presents a significant challenge in nuclear radiation measurements, particularly in neutron-gamma pulse shape discrimination, as it causes pulse distortion and diminishes identification accuracy. To address this, we propose an optimized Support Vector Regression (SVR) algorithm for correcting pulse pile-up. Initially, the Dung Beetle Optimizer (DBO) and Whale Optimization Algorithm (WOA) are integrated to refine the correction process, with performance evaluated using charge comparison methods (CCM) for pulse shape discrimination. Leveraging prior knowledge from simulated data, we further analyze the relationships between various types of pulse pile-ups, including their combinations, inter-peak distances, and the accuracy of corrections. Extensive experiments conducted in a mixed neutron-gamma radiation field using plastic scintillators demonstrate that the proposed method effectively corrects pulse pile-up and accurately discriminates between neutron and gamma. Moreover, our approach significantly improves the fidelity of pulse shape discrimination and enhances the overall reliability of radiation detection systems in high-interference environments.

## 1. Introduction

Pulse pile-up, a common phenomenon in radiation measurement, significantly depends on the detector type used. It occurs when multiple radiation events are registered within a very short time, often due to the limited temporal resolution of certain detectors. This issue is particularly pronounced under high-count-rate conditions and in noisy environments, leading to overlapping detector signals [1]. Such overlaps increase dead time, distort signals, and add complexity to data processing and interpretation [2]. In neutron-gamma pulse shape discrimination (PSD), addressing delayed fluorescence in pulses is crucial for accurate discrimination, as it highlights subtle differences in pulse decay profiles. Pulse pile-up, however, introduces distortions that hinder this accuracy, making the correction of distorted delayed fluorescence a primary focus in effective pile-up correction.

Traditional approaches typically utilize pulse pile-up rejection circuits to discard signals identified by hardware circuits as pile-up events [3,4]. However, these methods result in significant data loss and affect the accuracy of pulse discrimination. Furthermore, pile-up rejection circuits cannot completely prevent the transmission of overlapping pulses, leading to waveform distortion and spectral inaccuracies. Studies on pulse pile-up often overlaps with research on pulse waveform shaping algorithms [5,6,7]. Methods such as dual-exponential [8] and Gaussian functions [9] have been employed to model radiation pulses and their pile-ups. In addition, in the research on the activity estimation of gamma energy spectra, some researchers have also adopted the sparse reconstruction method to handle pulse pile-up [10,11,12]. For multiple pulse pile-ups, some researchers have also adopted an event-by-event modeling approach to handle them [13,14]. Researchers have constructed precise mathematical models of pulses to explore the underlying mechanisms of pulse pile-up and have used these models for effective pulse identification and correction [15,16]. Such modeling not only reveals the dynamic characteristics of pulse pile-up phenomena but also provides a theoretical foundation for advancing pulse processing technologies [17,18,19]. Extensive studies on the impact of pulse pile-up on pulse shape discrimination have demonstrated that pile-up can cause signal distortion, increased noise, complicate subsequent signal processing algorithms and lead to misidentification of particle types [20]. Benefiting from the rapid development of machine learning and artificial intelligence [21], advancements in waveform digitization technology have increasingly integrated high-speed ADCs [22,23] and FPGAs [24,25] into research on pulse pile-up and discrimination algorithms. A notable trend is the shift from traditional signal processing techniques to intelligent methods for addressing challenges in nuclear radiation detection [26,27]. For example, researchers such as Kafaee et al. [28,29] have utilized genetically optimized neural networks and sparse reconstruction methods to tackle pulse pile-up. Additionally, Liu and colleagues have introduced pulse-coupled neural networks into pulse processing algorithms, achieving discrimination of neutron-gamma pulses and demonstrating potential for handling pulse pile-up [30,31,32].

However, these studies often lack empirical validation and face issues of algorithmic complexity and limited generalizability. Support Vector Regression (SVR), developed from the principles of Support Vector Machines (SVM) in the 1990s by Vapnik and others [33], is a robust machine learning method with numerous applications in nuclear technology, including pulse shape discrimination and neutron spectrometry [34,35]. SVR operates by constructing a model that identifies an optimal hyperplane in high-dimensional space to minimize the error between actual and predicted pulse data, thereby enabling accurate distinction and correction of overlapped pulses. A significant challenge in applying SVR is the determination of the model’s optimal parameters, which is commonly addressed through various optimization techniques such as Grid Search Method (GSM) and cross-validation (CV).

To address the challenges of SVR parameter optimization, Meanwhile, in order to evaluate and address the pulse pile-up situation of the self-developed plastic scintillator detector and signal acquisition system, this paper introduces two novel algorithms, DBO-SVR and WOA-SVR, which are based on the Dung Beetle Optimizer (DBO) [36] and Whale Optimization Algorithm (WOA) [37]. These algorithms effectively correct pulse pile-up and improve the accuracy of neutron-gamma pulse waveform discrimination. Our key contributions are as follows:

(1) Inspired by the behavioral strategies of dung beetles and humpback whale, we design an DBO-SVR, aiming to avoid local optima of the model and enhance the global search capabilities of the optimization process, while eliminating the interference from complex backgrounds. In addition, we propose an WOA-SAR, which provides a robust global exploration capability for SVR parameter optimization, expressing excellent optimization, low overfitting risk and strong adaptability [38,39].
(2) We conduct a detailed classification of pulse pile-up and evaluate the proposed algorithms through both simulation and experimental methods. By leveraging prior knowledge from simulated data, we examine how various types of pulse pile-ups, their compositions, and peak-to-peak distances affect correction accuracy. The results indicate that peak-to-peak distance is the most significant factor influencing correction accuracy, with smaller distances leading to greater information loss due to signal overlap, thereby reducing the effectiveness of the correction process.
(3) Extensive experiments conducted in a mixed neutron-gamma radiation field using self-developed plastic scintillators detector and signal acquisition system demonstrate that both DBO-SVR and WOA-SVR effectively address the longstanding issue of pulse pile-up in nuclear radiation detection. Additionally, these methods pave the way for more reliable and efficient particle discrimination technologies. After completing the test of the algorithm, a preliminary understanding of the pulse pile-up situation of the entire detection system has been obtained, laying a foundation for the further integration of the algorithm in the future.

## 2. Theory and Experiment

### 2.1. Neutron-Gamma Pulse Shape Discrimination

In pulse shape discrimination, the electrical signals generated by neutrons and gamma rays in the detector exhibit notable differences. Gamma rays produce short, rapidly decaying pulse signals through ionization, whereas neutrons generate longer, more gradually decaying pulses via elastic scattering or other nuclear reactions. Discrimination methods typically employ time integration to capture these distinct pulse characteristics, comparing signals integrated over different time intervals to effectively distinguish neutrons from gamma rays.

**Figure 1:**
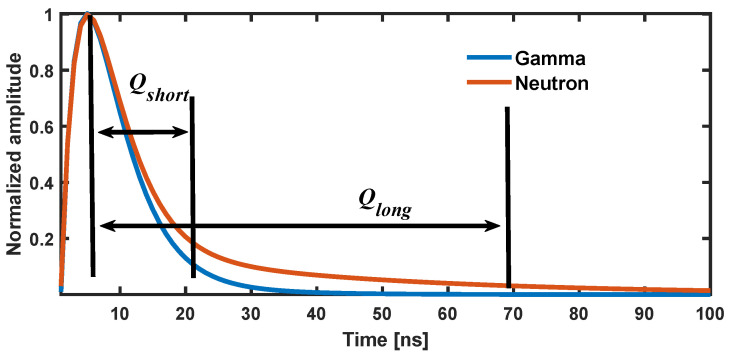
Schematic diagram of charge comparison method.

As shown in Figure 1, the graph illustrates the principle of the Charge Comparison Method (CCM) for discriminating between neutron and gamma-ray pulse shapes. This method differentiates neutrons and gamma rays by comparing the short-time integration ($Q_{\mathrm{short}}$) with the long-time integration ($Q_{\mathrm{long}}$) of the pulses. The time axis is in nanoseconds (ns), and the vertical axis represents the normalized amplitude, with the curves showing the time evolution of gamma-ray and neutron pulse shapes. The CCM quantifies these differences by calculating the integral values over short and long time intervals. A larger $Q_{\mathrm{short}}/Q_{\mathrm{long}}$ ratio generally indicates a gamma-ray pulse, while a smaller ratio typically corresponds to a neutron pulse.

### 2.2. Classification of Pulse Pile-Up

**Figure 2:**
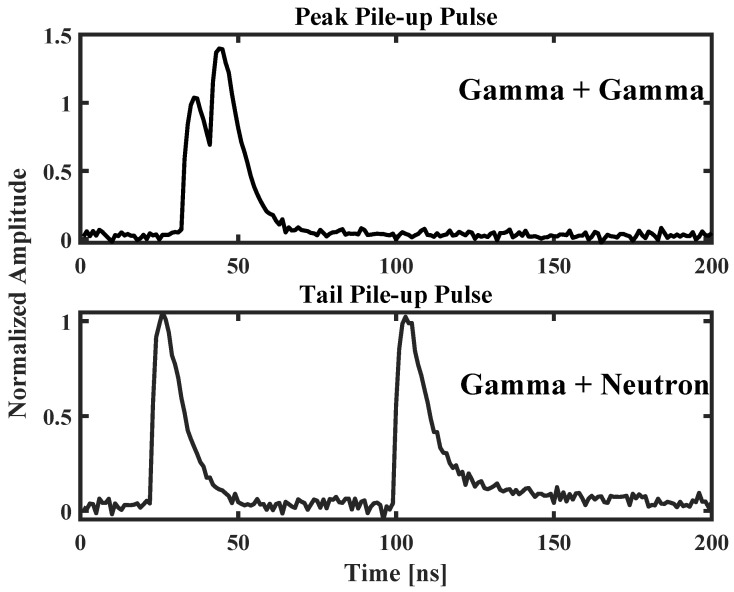
Different pile-up pulses of simulated data.

Pile-up pulses can be categorized into two types based on their effects on pulse amplitude: tail pile-up and peak pile-up. Tail pile-up refers to the occurrence where a subsequent pulse overlays the falling edge of a preceding pulse. Given that the falling edge of a pulse may persist for an extended period, tail pile-up can occur even under conditions of relatively low count rates [40]. Peak pile-up, the second type, happens when the interval between two adjacent pulses is too short, causing the neutron detection system to process these two pulses as a single pulse. A slight overlap results in a combined pulse with an amplitude slightly less than the sum of both. Peak pile-up not only leads to recorded spectral distortions, including accidental superimposed peaks, but also interferes with quantitative measurements made by assessing the full peak area of the energy spectrum. Figure 2 illustrates pulse pile-up simulated based on a mathematical model of the pulse, showcasing both types of pile-up scenarios.

Variations in formulations and the details of the preparation process can influence the pulse width of different plastic scintillators. Generally, the length of the falling edge of pulse waveforms from plastic scintillators is usually within 100–200 ns [41,42]. In this study, using self-prepared plastic scintillators, the measured pulse widths were within 100 ns. Therefore, it is considered that when the distance between the two peak values of a pile-up exceeds 100 ns, the pile-up does not affect the original information of the pulse. Corrections are necessary for pile-ups where the peak distance is less than 100 ns. Based on observations and definitions of pulse pile-up, when the peak distance exceeds 35 ns, the pulse can be classified as tail pile-up, otherwise, it is classified as peak pile-up, as illustrated in Figure 2, which shows both peak and tail pile-up scenarios.

In addition to classifying the type of pile-up, in mixed radiation fields where two or more types of particles are present, such as in an Am-Be source containing both gamma and neutron particles, the detection system may encounter several different scenarios of piled-up pulses [43]. If only two pulses are involved in the pile-up, there are four possible combinations: (1) γ + γ, (2) *n* + *n*, (3) γ + *n*, (4) *n* + γ, as shown in Figure 2, which depicts two scenarios of simulated pulse pile-ups. If three pulses are involved, there are eight possible combinations. However, in practical experiments, the probability of pile-ups involving three or more pulses is very low, thus, this study only considers scenarios where two pulses are piled up.

### 2.3. Preparation of Simulated Pulses

The empirical function for the pulse waveform response of organic scintillators to neutrons/gamma rays is generally a tri-exponential function with six parameters [44], as shown in the following equation:

(1) $$x\left(t\right)=A\left(e^{\frac{-(t-t_{0})}{\theta }}-e^{\frac{-(t-t_{0})}{\lambda_{s}}}+Be^{\frac{-(t-t_{0})}{\lambda_{l}}}\right)$$

where *A* represents the amplitude of the fast component at $t=t_{0}$, *B* represents the amplitude of the slow component at $t=t_{0}$, $\lambda_{s}$ is the decay constant for the fast component, $\lambda_{l}$ is the decay constant for the slow component, θ is the decay constant for the rise time, and $t_{0}$ is the reference time for the onset of the signal. To better approximate the actual characteristics of the pulses, this study incorporates the most significant delayed fluorescence component into the pulse shape discrimination model. In the empirical formula presented, the fast component represents the prompt fluorescence of the organic scintillator, while the slow component corresponds to the delayed fluorescence within the same scintillator. This study references the pulse characteristics of actual plastic scintillators, for individual γ/*n*, the simulated pulse signal duration is set to 200 ns, and the same applies to the length of pile-up pulses. The pulse information primarily covers 20 ns before the peak value and 180 ns after the peak value. To simulate more realistically, PMT dark current noise (μ = 0.03 δ = 0.02) [45] is added on top of the simulated pulses. The PMT in this study inherently produces dark current noise in the absence of light, due to thermal emissions, electronic fluctuations, and material impurities. This noise is characterized by its mean (μ = 0.03) and standard deviation (δ = 0.02). The mean defines the baseline noise level, while the standard deviation indicates its variability. A higher mean raises the noise floor, potentially masking low-intensity signals, while a larger standard deviation reflects greater fluctuations, affecting the stability and accuracy of weak signal detection.

### 2.4. Obtaining Experimental Pulses

In the pulse data acquisition experiment, this paper utilized a self-developed plastic scintillator with fast timing characteristics, primarily composed of Styrene, Methyl Methacrylate (MMA), 2,5-Diphenyloxazole (PPO), 9,10-Diphenylanthracene (DPA), and Divinylbenzene (DVB) [46,47]. These materials were prepared through an anaerobic thermal polymerization process. The preparation procedure is outlined as follows:

Styrene, MMA, and DVB monomers were individually passed through a chromatography column containing dry potassium carbonate on alkaline alumina to remove polymerization inhibitors. The materials, once free of inhibitors, were weighed, mixed, and the resulting solution was agitated using an ultrasonic device to ensure complete dissolution. The solution underwent vacuum treatment using a Schlenk line and was sealed in test tubes with a butane torch, creating a vacuum environment. The scintillator solution was then maintained at 60 °C for 24 h, with the temperature increasing by 5 °C daily until it reached 75 °C, where it was kept for 48 h until the plastic scintillator polymerized into shape. After polymerization, the test tubes were slowly cooled to room temperature to reduce internal stress, then broken and polished to obtain the plastic scintillator.

**Figure 3:**
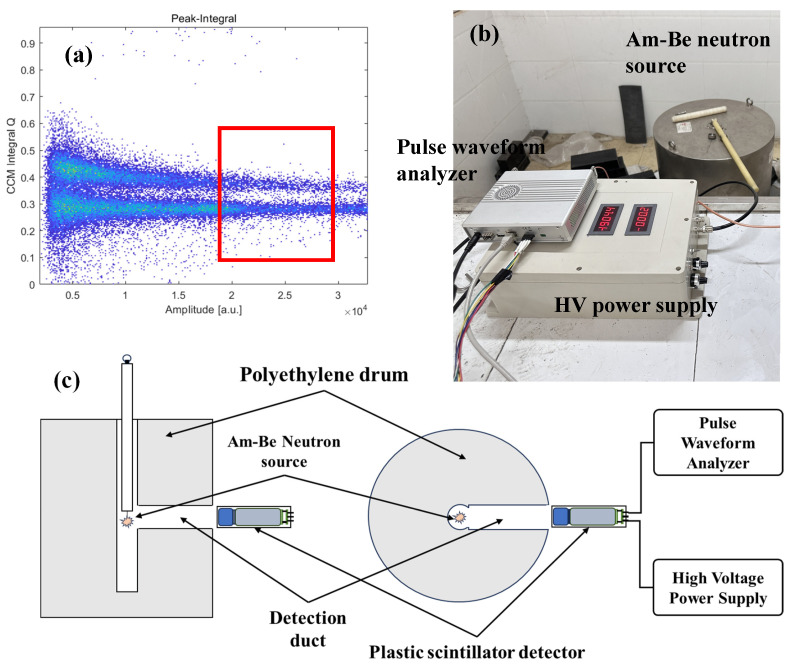
Acquisition of experimental data, (**a**) Pulse discrimination scatter plot obtained through CCM, (**b**) Neutron source chamber measurement, (**c**) Schematic diagram of pulse acquisition experiment.

The plastic scintillator was coupled to a photomultiplier tube, ET 9821KB (ET Enterprises, Ltd., Uxbridge, UK), using silicone oil. Pulse data collection was performed using a high-speed, self-developed pulse signal acquisition system with a sampling rate of 1 GSPS and a resolution of 16 bits. An Am-Be neutron source was used as the radiation source. As shown in Figure 3, the neutron-gamma pulse signal acquisition experiment took place at the Neutron Radiation Source Room of the College of Nuclear Technology and Engineering at Chengdu University of Technology. The radiation source was placed inside a polyethylene drum, during measurements, it was lifted from the bottom to the mouth of the bore, and the detector was positioned at the entrance of the bore.

As shown in Figure 3a, the scatter plot obtained by the CCM for neutron-gamma discrimination is displayed, where larger integral values represent neutron pulse signals and smaller values indicate gamma pulse signals. The section marked with a red box highlights the individual neutron/gamma training pulse data used in the pulse pile-up correction experiment, with a detailed discussion provided in Section 3. Figure 3b shows the experimental setup, and Figure 3c illustrates a schematic of the experimental arrangement.

### 2.5. SVR and Its Optimization Algorithm Principles

The SVR model is effective in handling nonlinear regression problems, providing a mechanism for continuous numerical prediction by finding a linear function in a high-dimensional space that minimizes the prediction error. Below, the principle of SVR for pulse pile-up correction is described [48,49].

The pulse sample sequence is *D*:

(2) $$D=\left\{\left(x_{i},y_{i}\right)|i=1,2,...,m\right\}$$

Among them, $x_{i}$ is the input sample and $y_{i}$ is the actual output. The regression function in high-dimensional space is as follows:

(3) $$f\left(xi\right)=\omega^{T}\varphi \left(xi\right)+b$$

where ω and *b* are the parameters to be determined. To find ω and *b*, the optimization problem is defined as follows:

(4) $$\min\frac{1}{2}\left||\omega \right|{|}^{2}+C\cdot \sum_{i=1}^{m}\left(\xi i+\hat{\xi }i\right)$$

(5) $$\begin{array}{c} \begin{array}{c} s,t.\left\{\begin{array}{c} y_{i}-f\left(x_{i}\right)\leq \varepsilon +{\hat{\xi }}_{i}\\ f\left(x_{i}\right)-y_{i}\leq \varepsilon +\xi_{i}\\ \xi_{i},{\hat{\xi }}_{i}\geq 0,\left(i=1,2,...,m\right) \end{array}\right. \end{array} \end{array}$$

The regression function is obtained as follows:

(6) $$f\left(x\right)=\sum_{i,j=1}^{m}\left(\alpha i-\hat{\alpha }i\right)K\left(xi,xj\right)+b$$

where $\alpha_{i}$ and $\hat{\alpha }i$ are Lagrange multipliers, and $K\left(xi,x_{j}\right)$ is the kernel function.

**Figure 4:**
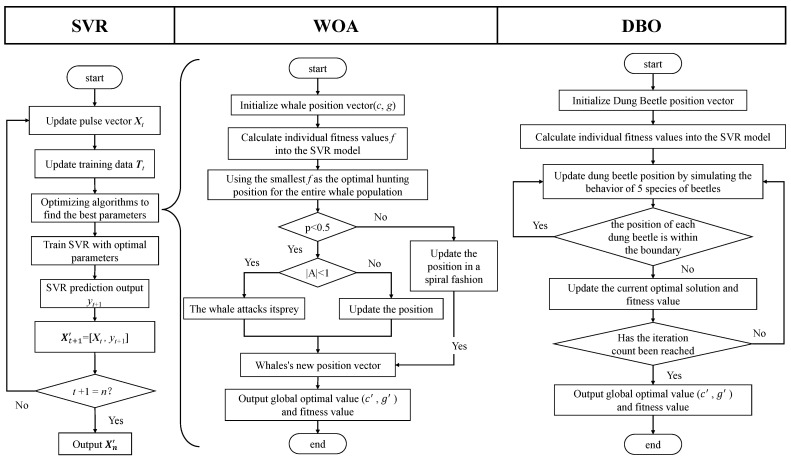
Flowcharts for SVR, DBO-SVR, and WOA-SVR.

In this paper, two optimization algorithms, DBO and WOA are employed for parameter optimization to enhance the performance of the SVR model, particularly for tasks such as time series prediction. These algorithms focus on optimizing key parameters of SVR, namely the penalty parameter *C* and the kernel parameter γ. The processes for both algorithms are depicted in Figure 4.

In Figure 4, *n* represents the number of periods to be predicted, $X_{t}$ represents the pulse vector to be predicted with a length of *t*, $X_{t}^{\prime }$ represents predicted pulse vector at time *t*, $y_{t+1}$ represents predicted the pulse value at time t+1, $X_{t+1}^{\prime }$ represents the predicted the pulse vector at time t+1, (*c*, *g*) is the optimal parameter value of SVR obtained by the optimization algorithm.

DBO is an algorithm that simulates various behaviors of dung beetles in nature to solve optimization problems. For a detailed derivation and principles, please refer to the literature [36]. The algorithm primarily updates the position of dung beetles through the following behaviors:

(1) Rolling behavior: Simulates dung beetles rolling dung balls in a straight line using celestial cues, adjusting their position by modifying the deviation coefficient k and a random factor α to approach the globally worst position $X_{\omega }$, thereby mimicking the effect of varying light intensities. (2) Dancing behavior: When encountering obstacles, dung beetles adjust their direction through a dancing motion, modeled by a tangent function, to find new paths. (3) Breeding behavior: Dung beetles roll dung balls to safe areas suitable for laying eggs. As iterations progress, a reduction factor R is used to dynamically adjust the boundaries of the breeding area to accommodate local optima $X^{*}$. (4) Foraging behavior: Establishes an optimal foraging area where young dung beetles update their positions based on the global best position $X_{b}$ and the boundaries of the active area. (5) Stealing behavior: Some dung beetles attempt to steal resources from others, updating their position using a random vector *g* and a constant *S*.

WOA is a meta-heuristic optimization algorithm that simulates the hunting behavior of humpback whales. For a detailed derivation, refer to the literature [37]. The primary process is as follows: (1) Encircling prey mechanism: Whales update their positions by reducing the control parameter *a* from 2 to 0 and using random vectors A fluctuating within [−a, *a*] and *C*, thus approaching the current best solution. (2) Spiral updating mechanism: Utilizes the logarithmic spiral equation, calculating the distance $D_{2}$ between the whale and the target, combined with constant *b* and random number *l*, to simulate the whale’s spiral movement pattern. Additionally, when the exploration parameter |A| exceeds 1, whales no longer approach the current best solution but update their position based on a randomly selected whale from the population. This enhances the global search capability and maintains population diversity. Through these strategies, WOA effectively balances global exploration and local exploitation, enhancing the probability of finding the global optimum.

## 3. Results and Discussion

Prior to pile-up correction, it is imperative to standardize the pulse data. All pulse data are standardized to a duration of 200 ns. The training data consist of individual gamma/neutron pulses, with each pulse selected from the interval of 20 ns before the peak to 180 ns after the peak. The pile-up pulses are selected from the interval of 20 ns before the peak of the first pulse to 180 ns after the peak. In practical neutron-gamma discrimination radiometric applications, the occurrence of three or more pulse pile-ups is exceedingly rare. Therefore, this article primarily addresses the correction for the case of two-pulse pile-up.

In the correction of simulated/experimental pulse pile-up, this study selected 5000 pulses for training, with 500 designated as validation pulse. The mean squared error (MSE) of the validation pulse data was calculated and compared across three algorithms, as presented in Table 1. For simulated data, given its richer a priori knowledge, this paper compared the accuracy of pile-up correction and its relationship with the spacing between two pulse peaks. Additionally, the overall effectiveness of pile-up correction was assessed using the CCM. For experimentally measured pile-up pulse data, lacking prior knowledge, the evaluation of pile-up correction effectiveness was primarily conducted through the CCM value and the post-discrimination Figure of Merit (FoM) value.

### 3.1. Correction Results of Simulated Pile-Up Pulses

**Figure 5:**
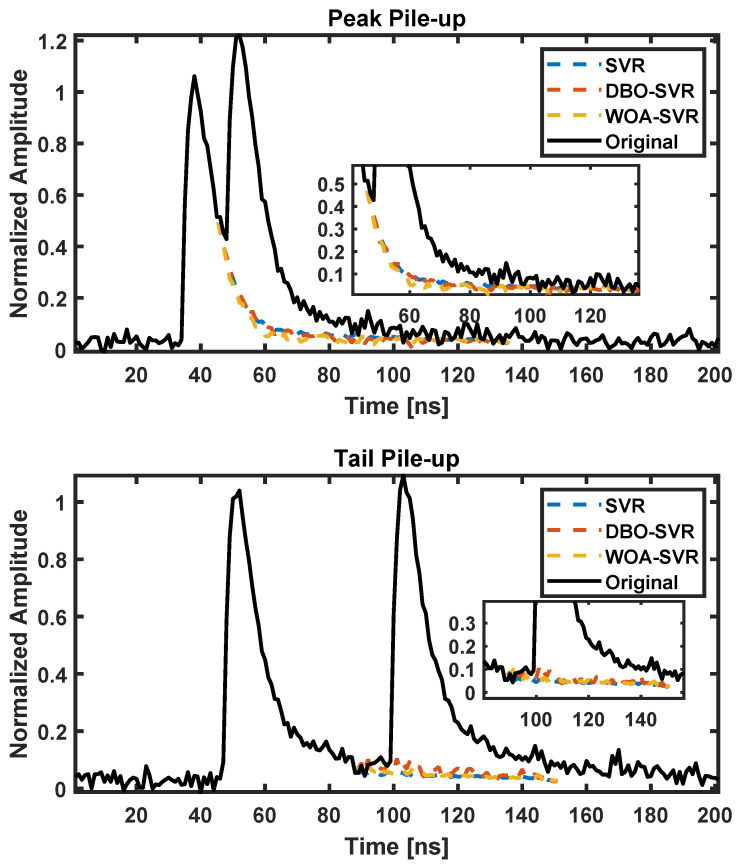
Pulse pile-up correction results for different SVR algorithms based on simulated data.

As shown in Figure 5, the correction results of two types of pile-up based on simulated data are displayed.

From the graph, it can be seen that all three methods effectively completed the prediction and correction of pulse pile-up, filling in the missing parts of the pulse’s falling edge. Upon closer examination, the pulses predicted by SVR are smoother, and the overall pulse height reduction is more significant than the other two optimized SVR algorithms, resulting in the loss of more detail. This is an indication of SVR’s tendency to fall into local optima.

**Figure 6:**
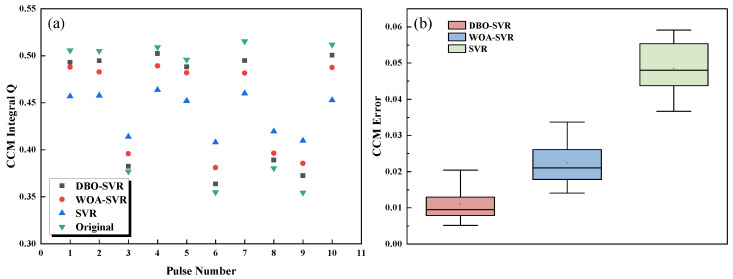
CCM values and error bars for the test dataset, (**a**) Randomly selected 10 pulses and predicted using three methods to compare CCM values, (**b**) The CCM error bars of three predicted method.

High accuracy in pulse shape is essential for effective neutron-gamma pulse shape discrimination. When processing pulses, it is crucial to restore the original pulse shape accurately. Additionally, CCM provides a comprehensive assessment of pulse shape quality, further supporting discrimination accuracy. As shown in Figure 6, the CCM values and their error bars from simulated test data indicate that DBO-SVR restores pulses best, with the least error in CCM values. Additionally, it was noted that the CCM values are generally lower with SVR predictions than the original pulses, suggesting that the predicted pulse’s falling edge is lower and more prone to local optima.

**Figure 7:**
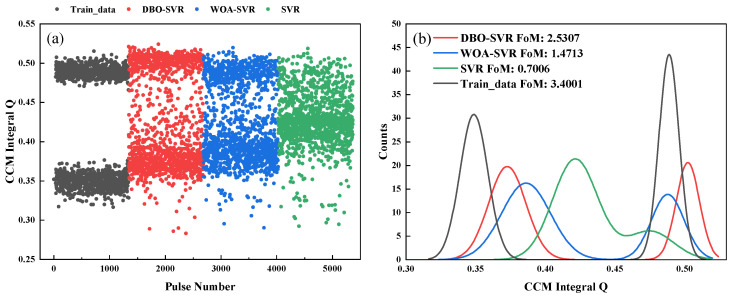
Scatter CCM values and FoM values for three methods based on simulated data. (**a**) Scatter plots of the three methods and the training data, (**b**) FoM values for three methods.

Figure 7a shows a scatter plot for discrimination, and Figure 7b presents statistical FoM figures. The final FoM figure indicates that the information loss caused by pulse pile-up is difficult to restore, with DBO-SVR providing the best pulse correction effect, and SVR barely able to distinguish between the two types of pulse particles.

**Figure 8:**
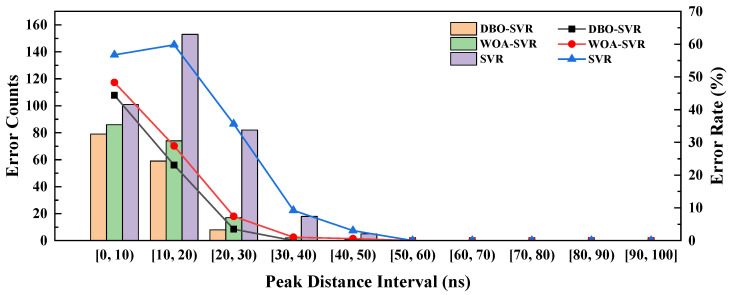
Restoration of pulse pile-up by three methods, error counts, and error rates.

For simulated pile-up pulses, a lot of prior knowledge can be used to statistically analyze the correction status. This study documented the impact of peak-to-peak distances on pulse pile-up correction outcomes, as shown in Figure 8, where shorter peak-to-peak distances correlated with higher error rates. Tail pile-ups showed better correction outcomes than peak pile-ups.

Table 2 presents the correction statistics for four different types of pulse pile-up combinations, corrected by three distinct algorithms. For the γ + γ combination, WOA-SVR and DBO-SVR demonstrate superior performance compared to SVR, particularly noticeable in the 10–20 ns to 30–40 ns intervals where their corrections are closest to the total occurrences. Both algorithms manage to sustain high correction rates up to the 80–90 ns interval, showcasing robust consistency in handling γ + γ pile-ups.

In *n* + *n* type pile-ups, WOA-SVR and DBO-SVR also exhibit a notably high correction performance, particularly in the 0–10 ns to 30–40 ns intervals, aligning closely with the total occurrences. For this type of pile-up, the overall accuracy of pulse prediction has decreased, especially in the 0–30 ns interval. When the pulse distances is greater than 30 ns, the pulse prediction is completely correct.For the mixed-type pile-ups (γ + *n* and *n* + γ), DBO-SVR and WOA-SVR show a more consistent correction capability across all intervals, particularly excelling in the 20–30 ns to 40–50 ns range for γ + *n* combinations. These algorithms efficiently address the complexities introduced by the heterogeneity of gamma and neutron pile-ups. In general, the performance of DBO-SVR and WOA-SVR is generally more consistent and robust across all types of pile-ups.

However, regardless of the method employed, the correction accuracy for pile-up pulses initiated by neutron pulses, particularly in the 0–10 ns interval, shows a noticeable decline. This decrease may be attributed to the fact that the distinction between neutron and gamma pulses in the 0–10 ns range is not sufficiently pronounced, causing the algorithms to fall into a local optimum. The gradient of gamma pulse decay is steeper, leading the predicted pulse points to fall in the direction of the steeper gradient. Consequently, more gamma pulses are incorrectly predicted in this interval.To address this issue, it is necessary to incorporate additional characteristic information of neutron and gamma pulses to enhance the accuracy of pile-up pulse correction predictions. Further research will continue in this area in future work.

### 3.2. Correction Results of Experiment Pile-Up Pulses

**Figure 9:**
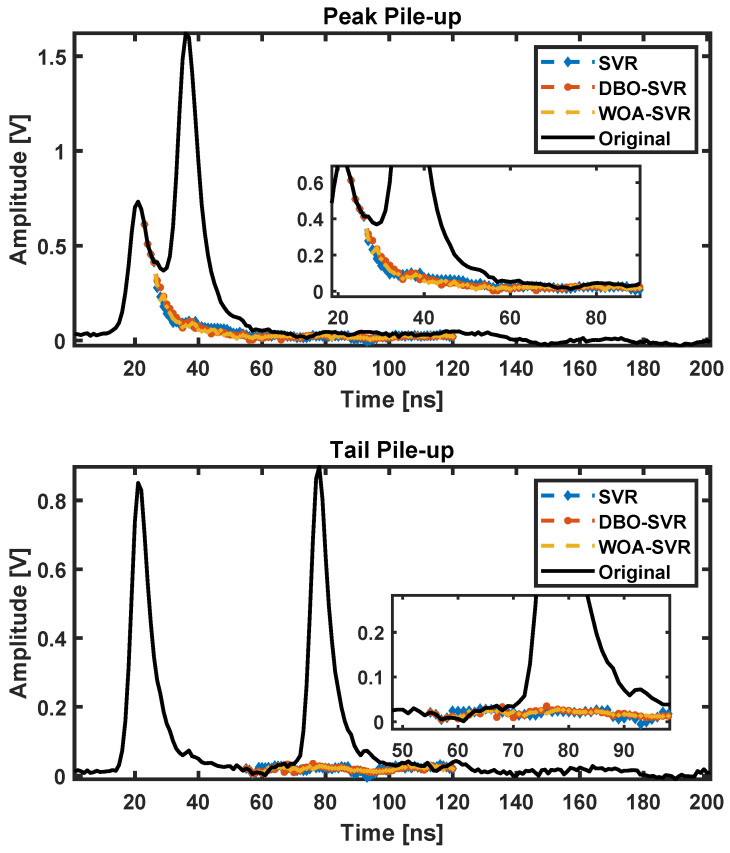
Pulse pile-up correction results based on plastic scintillators using different SVR methods.

The pulse pile-up correction results based on plastic scintillators as shown in Figure 9. The inset zooms into the critical region highlighting how each algorithm predicts the waveform. DBO-SVR and WOA-SVR show a closer approximation to the original waveform’s tail after the main peak compared to SVR, suggesting they might be preserving the waveform integrity more effectively while minimizing the peak distortion caused by the pile-up. In the trailing edges of these peaks, DBO-SVR and WOA-SVR demonstrate a more effective reduction in the tailing artifacts, maintaining waveform shape more faithfully compared to SVR, which shows slight deviations from the original waveform. Overall, the DBO-SVR and WOA-SVR algorithms appear to perform better in correcting both types of pile-ups, effectively reducing the distortions and preserving the original waveform’s characteristics more closely than SVR.

**Figure 10:**
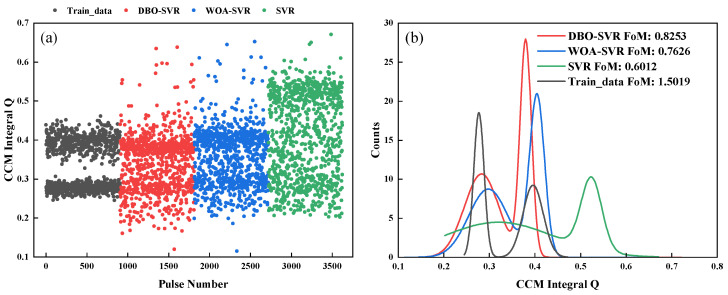
Scatter CCM values and FoM values for three methods after correction based on experimental data. (**a**) Scatter plots of the three methods and the training data, (**b**) FoM values for three methods.

As shown in Figure 10, the CCM results after correction by the three methods show that DBO-SVR again demonstrated the best pile-up correction outcomes. Compared to DBO-SVR and WOA-SVR, the SVR scatter plot was broader and did not exhibit a clear convergence trend, indicating significant improvements in training accuracy and prediction precision after the addition of optimization algorithms to the SVR model.

### 3.3. Comparison Before and After Correction

**Figure 11:**
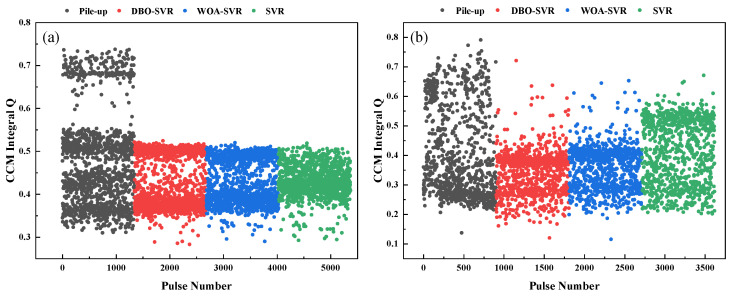
Comparison of scatter plots before and after correction, (**a**) simulated data, (**b**) Experimental data.

Figure 11 illustrates the effectiveness of different pulse pile-up correction algorithms, distinguishing between simulated data in Figure 11a and experimental data in Figure 11b, based on CCM values across various pulses. In Figure 11a, the data points are grouped into four distinct categories representing the simulated pile-ups before correction and the results after applying three different correction algorithms: SVR, DBO-SVR, and WOA-SVR. The uncorrected pile-up data points, shown in gray, are dispersed widely, reflecting substantial variability in CCM values due to overlapping pulses. In Figure 11b, which displays experimental data, a clearer improvement in data segregation is evident after correction. The pulses corrected by DBO-SVR, WOA-SVR, and SVR form more compact clusters compared to the pre-correction scenario. This improvement suggests that each correction method effectively reduces variability and aligns the CCM values more consistently, with DBO-SVR showing particularly tighter clustering. This indicates a superior ability of DBO-SVR to mitigate the effects of pulse overlap, preserving the integrity of pulse information more effectively than WOA-SVR and SVR. This distinction between simulated and experimental data highlights the practical application and validation of these algorithms in real application.

## 4. Conclusions and Outlook

In nuclear radiation detection, pulse pile-up is unavoidable, particularly under conditions of high count rates and high noise levels. With the advancement of digital pulse waveform technology and intelligent pulse processing methods, significant progress has been made in pulse pile-up correction research. This paper proposes a pulse pile-up correction strategy based on time-series prediction, using support vector regression (SVR) and its optimized algorithms (DBO-SVR and WOA-SVR). Furthermore, it explores the role of these correction methods in neutron-gamma pulse shape discrimination.

This paper first categorizes types of pulse pile-up and evaluates correction algorithms through both simulation and experimental approaches. Leveraging prior knowledge from simulated data, it examines how variations in pile-up types, compositions, and peak-to-peak distances affect correction accuracy. Results indicate that peak-to-peak distance has the greatest impact on accuracy; shorter distances lead to increased information loss due to pile-up, thus diminishing correction accuracy. Specifically, corrections for tail pile-ups achieve higher accuracy than those for peak pile-ups. The study also quantifies the accuracy of corrections across various pile-up compositions and inter-peak distances, finding that gamma-gamma (γ + γ) pile-up corrections yield the highest accuracy, while neutron-gamma (*n* + γ) corrections exhibit the lowest. SVR models optimized with DBO and WOA algorithms show substantially higher accuracy in restoring original pulse shapes compared to conventional SVR. Furthermore, the advanced SVR models not only effectively correct pulse pile-up but also enhance neutron-gamma pulse discrimination accuracy. CCM and FoM analyses reveal that DBO-SVR, in particular, excels in preserving pulse shape integrity, resulting in more accurate particle identification.

While this paper presents substantial advancements, it also identifies areas for further investigation. The research primarily focuses on two-pulse pile-ups, given their high occurrence probability in practical scenarios. However, as radioactive source activity increases, pulse pile-up becomes more frequent and complex, with a higher likelihood of three or more pulses overlapping. This complexity was not fully addressed in the present study. Future work will aim to enhance the algorithm to handle such scenarios, expanding its applicability across a broader range of detection systems and experimental conditions. Additionally, applying the proposed models to different types of scintillators and radiation sources could demonstrate their versatility and broader applicability. Furthermore, incorporating real-time processing capabilities into the SVR models would enhance their practical utility. Implementing these models on high-speed data acquisition systems with advanced hardware, such as FPGAs, could facilitate real-time pulse pile-up correction, significantly boosting the efficiency of radiation detection systems in dynamic environments.

Additionally, this paper provides only a preliminary description of delayed fluorescence in the simulation experiments, using CCM to evaluate the algorithm’s performance in reconstructing delayed fluorescence, without an in-depth discussion. Integrating delayed fluorescence—an important parameter in discrimination—into the model may improve pulse pile-up correction. Due to time and resource limitations, we plan to investigate this approach in future work to enhance the algorithm’s general applicability.

## Figures and Tables

**Table 1:** Mean MSE of simulation pulse and plastic scintillator.

	SVR	DBO-SVR	WOA-SVR
Simulation Data	$5.41\times 10^{-4}$	$3.25\times 10^{-4}$	$3.75\times 10^{-4}$
Plastic Scintillator	$3.10\times 10^{-4}$	$3.02\times 10^{-4}$	$2.26\times 10^{-4}$

**Table 2:** Correct correction statistics for four different pile-up combinations and distances.

Type	γ+γ									
Distance (*ns*)	0–10	10–20	20–30	30–40	40–50	50–60	60–70	70–80	80–90	90–100
Total	56	75	63	63	49	47	21	23	10	0
SVR	5	11	31	54	45	47	21	23	10	0
WOA-SVR	35	53	59	63	48	47	21	23	10	0
DBO-SVR	31	55	63	63	49	47	21	23	10	0
Type	n+n									
Distance (*ns*)	0–10	10–20	20–30	30–40	40–50	50–60	60–70	70–80	80–90	90–100
Total	33	55	42	31	26	32	15	15	4	0
SVR	14	35	36	31	26	32	15	15	4	0
WOA-SVR	17	35	36	31	26	32	15	15	4	0
DBO-SVR	29	39	36	31	26	32	15	15	4	0
Type	γ+n									
Distance (*ns*)	0–10	10–20	20–30	30–40	40–50	50–60	60–70	70–80	80–90	90–100
Total	45	57	67	52	45	33	20	21	3	0
SVR	3	8	23	43	44	33	20	21	3	0
WOA-SVR	26	49	60	50	45	33	20	21	3	0
DBO-SVR	27	53	65	52	45	33	20	21	3	0
Type	n+γ									
Distance (*ns*)	0–10	10–20	20–30	30–40	40–50	50–60	60–70	70–80	80–90	90–100
Total	44	69	58	49	44	31	25	15	4	0
SVR	6	23	18	24	43	31	25	15	4	0
WOA-SVR	19	34	35	24	43	31	25	15	4	0
DBO-SVR	23	38	41	24	43	31	25	15	4	0

## Data Availability

Data are contained within the article.
